# Idiopathic nodular glomerulosclerosis and differential diagnosis

**DOI:** 10.1590/2175-8239-JBN-2019-0229

**Published:** 2020-08-07

**Authors:** Sergio Raúl Alvizures Solares, Héctor Raúl Ibarra-Sifuentes, María Guadalupe Ramírez Ramírez, Giovanna Yazmin Arteaga Muller, Jesús Cruz Valdez

**Affiliations:** 1 Universidad Autónoma de Nuevo León, University Hospital, Internal Medicine Department, Monterrey, México.; 2 Universidad Autónoma de Nuevo León, Hospital Universitario, Servicios Clínicos Nefrología, Monterrey, México.

**Keywords:** Diabetic Nephropathies, Biopsy, Hypertension, Tobacco Use Disorder, Nefropatias Diabéticas, Biópsia, Hipertensão, Tabagismo

## Abstract

**Introduction:**

Idiopathic nodular glomerulosclerosis (ING) is a condition that has a vasculopathic glomerular histological pattern.

**Case presentation:**

The authors present the case of a 44-year-old Hispanic smoker female with hypertension and peripheral arterial disease who presented nephrotic syndrome for 2 weeks. The patient was diagnosed with ING by percutaneous renal biopsy results, which showed global nodular mesangial matrix expansion, with linear staining accentuation of glomerular and tubular basement membrane for Immunoglobulin G (IgG) and albumin on immunofluorescence.

**Conclusions:**

ING is a rare disease with a poor renal prognosis and wide diagnostic approach; we highlight the importance of analyzing every piece of detail together to reach a definitive diagnosis.

## RESUMO

**Introdução:**

A glomerulosclerose nodular idiopática (GNI) tem um padrão histológico glomerular vasculopático.

**Apresentação do caso:**

Os autores apresentam o caso de uma mulher latino-americana, de 44 anos, fumante, com hipertensão e doença arterial periférica; com síndrome nefrótica por 2 semanas. Ela foi diagnosticada com GNI por biópsia renal percutânea, que mostrou expansão generalizada da matriz mesangial nodular, com acentuação de coloração linear na membrana basal glomerular e tubular para imunoglobulina G (IgG) e albumina à imunofluorescência.

**Conclusões:**

A GNI é uma doença rara, com mau prognóstico renal, e com necessidade de uma ampla abordagem diagnóstica. Demonstramos aqui a importância de se analisar todos os detalhes em conjunto para realizar um diagnóstico definitivo.

## INTRODUCTION

Idiopathic nodular glomerulosclerosis (ING) is a rare condition that has vasculopathic glomerular histological pattern, which represents 0.45% of all the biopsies in large series^1^. Alpers and Biava were the first to report this new entity, and a year later Herzenberg et al. adopted the ING terminology.

## CASE REPORT

**Figure 1:**
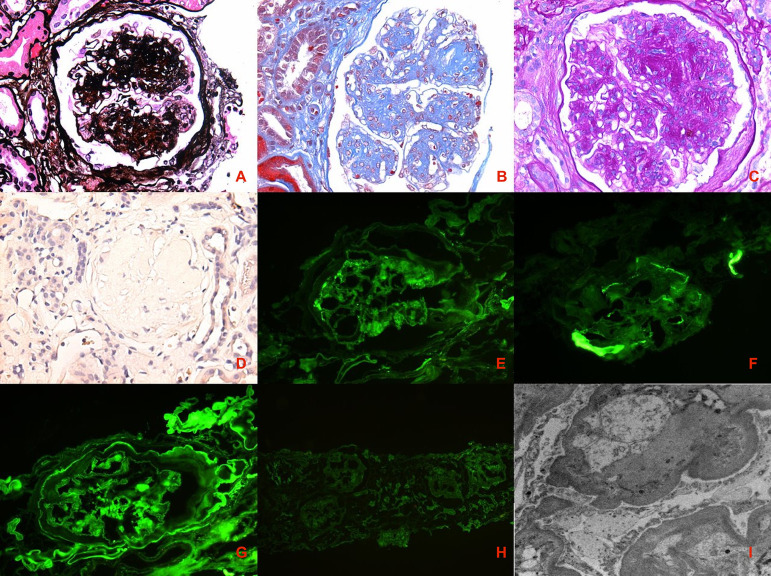
Histological findings of a patient with smoking-modified hypertension-associated nodular glomerulosclerosis. Panel A shows silverpositive mesangial matrix expansion with nodular appearance and prominent GBM (Silver Jones stain, 40×). Panels B and C show mesangial matrix expansion with nodular appearance and prominent GBM (Trichrome Masson staining and Periodic Acid Schiff staining, respectively, 40×). Panel D shows 40x negative Congo Red staining with negative birefringence (not shown). Panels E, F, G, and H show linear accentuation of GMB and TMB (Kappa, Lambda, IgG and Albumin Immunofluorescence, respectively, 40×). Panel I, electron microscopy image shows increase in GMB thickening, average 900 nm, and diffuse foot process effacement. GMB: glomerular basement membrane; TMB: tubular basement membrane; IgG: Immunoglobulin G.

A 44-year-old woman who was current smoker (25 packs/year), with hypertension and peripheral arterial disease diagnosed 2 and 1 years ago, respectively, presented with asthenia, nausea, and vomiting during the previous 2 weeks. The physical examination was unremarkable, except by blood pressure of 140/70 mmHg, body mass index of 22.1 kg/m^2^, and pallor. Relevant laboratory findings were hemoglobin of 7 gr/dL, creatinine of 4 mg/dL, albumin 3.5 gr/dL, sodium 128 mEq/L, potassium 4.5 mEq/L, hypercholesterolemia (244 mg/dL, reference value >200 mg/dL), and proteinuria of 7.4 grams in 24 hours. Anti-nuclear antibodies, anti-glomerular basement membrane antibodies, anti-neutrophil cytoplasmic antibodies, human immunodeficiency virus, hepatitis B and C virus tested negative. Renal ultrasound showed normal sized kidneys without obstruction. Percutaneous renal biopsy (Figure 1) was performed demonstrating 27 glomeruli, of which 23 were globally sclerosed, with global nodular mesangial matrix expansion, which stained blue with the Masson trichrome, and was Congo Red negative by light microscopy (LM). By Immunofluorescence (IF), there was no immune deposit, except by the linear staining accentuation of glomerular basement membrane (GBM) and tubular basement membranes (TBM) for Immunoglobulin G and albumin. By electron microscopy (EM), glomerular basement membranes thickness was increased, with laminated and healed areas. There was >50% of interstitial fibrosis and proportional tubular atrophy. Arterioles showed moderate hyalinosis and interlobular arteries mild media thickening. Analyzing the data of the medical history, physical examination, and laboratory and histological findings, ING was diagnosed.

## DISCUSSION

The pathogenesis of ING has been associated with heavy longstanding cigarette smoking and uncontrolled hypertension^1^^,^ 2. Several observations demonstrated an overexpression of glomerular advanced glycation end-products and their receptor, suggesting that this system is activated, leading to ING^3^. Probably, this entity represents a form of hypertensive glomerulosclerosis altered by smoking-derived products; thus, the adequate term for the condition is smoking-modified hypertension-associated nodular glomerulosclerosis (SHaNGS).

SHaNGS mainly affects older (68.2 years) men (78.2-80%, male:female ratio of 4:1), and the clinical presentation reassembles renal insufficiency and nephrotic range proteinuria in 82 and 69% of the cases, respectively^1^^,^ 4. Around 96% of the patients have 15 years mean duration of history of hypertension, 91% has a history of smoking with a mean cumulative intake of 52.9 pack-years and 90% has a history of hypercholesterolemia¹.

The differential diagnosis of nodular glomerulopathies is wide and includes membrano-proliferative glomerulonephritis (MPGN), monoclonal immunoglobulin deposition disease, amyloidosis, fibrillary, immunotactoid glomerulonephritis, collagen type III disease, thrombotic microangiopathy, and chronic hypoxic or ischemic conditions^1^^,^ 4 ^-^ 6(Table 1); the distinction between them requires an integrative approach, involving clinical history, serological markers, and histology findings of LM, IF, and EM.

**Table 1.** Differential diagnosis of nodular glomerulosclerosis.

Disease	Clinical	LM	IF	EM
Advance Diabetic Nephropathy	DM (+).	TMB and GBM thickening, mesangial expansion, non-isometric Kimmelstiel-Wilson nodules, afferent and efferent arterioles hyalinosis.	Negative. Linear accentuation of GBM and TMB for IgG and albumin.	Mesangial matrix and cellularity increase, diffuse TMB and GBM thickening, diffuse foot process effacement.
Membrano-proliferative Glomerulonephritis	Etiology dependent.	Endocapillary proliferation, lobular accentuation, diffuse GBM thickening with double contour.	Diffuse granular GBM and mesangial staining of IgG (polyclonal or monoclonal); Dominant or only C3.	Mesangial and subendothelial granular deposits.
ING or SHaNGS	Smoking and Hypertension, DM History (-).	Mesangial matix expansion with nodular appearance, GBM thickening, arteriolar hyalinosis and glomerulomegaly.	Negative. Linear accentuation of GBM and TMB for IgG and albumin.	Negative. Linear accentuation of GBM and TMB for IgG and albumin.
Amyloidosis	Etiology dependent.	Acellular, amorphous, pale-pink material in mesangium, GBM, interstitium and arteries. Congo Red, positive apple-green birefringence.	Restricted monoclonal light chain staining.	Randomly oriented, non-branching, straight fibrils (8–12 nm in diameter) and foot process effacement.
Fibrillary Glomerulonephritis	Unknown etiology.	Nodular and membrano-proliferative appearance. Congo red-negative.	Polyclonal IgG (IgG4) and C3 staining.	Randomly oriented, non-branching, straight fibrils (12–24 nm in diameter) in mesangium and GBM.
Immunotactoid Glomerulonephritis	Nodular and membrano-proliferative appearance. Congo red-negative.	Nodular and membrano-proliferative appearance. Congo red-negative.	Monoclonal IgG with kappa or lambda light chain staining.	GBM and mesangial microtubular deposits in parallel arrays (>30 nm in diameter).
GBM and mesangial microtubular deposits in parallel arrays (>30 nm in diameter).	Paraprotein in blood and/or urine.	Mesangial expansion with nodular appearance.	Restricted monoclonal linear light and/or heavy chain staining in mesanguim, TMB and GBM.	Finely granular or “pepper-like” appearance deposits in TMB outer part and GBM inner part.
Type III Collagen Glomerulopathy	Blood and urine N-terminal procollagen type III peptide.	May be hypercellular.	Negative.	Curved fibers with 60 nm periodicity.
Fibronectin Glomerulopathy	Family history.	PAS positive mesangial deposits.	Negative, except for fibronectin staining.	Massive electron-dense deposits in mesangial matrix.
Chronic Cyanotic or Ischemic Conditions	Etiology dependent.	Centrolobular mesangial thickening, hyaline mosaic pattern deposition, mesangiolytic lesions, glomeruli microaneurysms and arterioles hyaline deposition.	Negative	Intramembranous and mesangial electron-dense deposits.

LM: Light Microscopy; IF: Immunofluorescence; EM: Electron Microscopy; DM: Diabetes Mellitus; GBM: Glomerular basement membrane; TMB: Tubular basement membrane; ING: Idiopathic Nodular Glomerulosclerosis; SHaNGS: Smoking modified Hypertension associated Nodular Glomerulosclerosis; IgG: Immunoglobulin G; C3: Complement C3; PAS: Periodic acid-Schiff; IgM: Immunoglobulin M.

SHaNGS differs from diabetic nodular glomerulosclerosis only by clinical background; also, SHaNGS presents with severe extrarenal vascular disease^1^^,^ 7. The histology of SHaNGS by LM shows global and diffuse mesangial matrix expansion with focal sclerosis nodular formation and glomerulomegaly with lobular appearance^1^. Interestingly, mesangial nodules contain increased endothelial-lined vascular spaces, suggesting neovascularization^1^. The non-atrophic tubules show TBM thickening, evoking the changes in diabetic glomerulosclerosis^1^. Arteriosclerosis and arteriolosclerosis with hyalinosis is a prominent finding in all cases^1^. By IF, there is no documented immune deposits, except by the linear staining accentuation of GBM and TBM for immunoglobulin G and albumin in half of the cases^1^. By EM, all cases show prominent mesangial sclerosis and GBM thickening, with a mean of 926 ±46.5 nm; with foot process effacement in 46% of patients^1^.

In the present case, the absence of immune deposits by IF excluded MPGN and dysproteinemic-related disease; in addition, there was no electrodense deposits by EM, suggesting SHaNGS diagnosis. The authors reviewed the clinical history and the patient had no diabetes mellitus diagnosis; also, oral glucose tolerance and glycosylated hemoglobin ruled out diabetes mellitus. According to Markowitz et al. criteria^1^, SHaNGS was diagnosed by the absence of clinical evidence of DM, the histological finding of nodular mesangial sclerosis, and the exclusion by IF and EM of chronic membranoproliferative glomerulonephritis, chronic thrombotic microangiopathy, amyloidosis, monoclonal immunoglobulin deposition disease, fibrillary glomerulonephritis, and glomerulonephritis.

Treatment for SHaNGS consists on aggressive management of blood pressure with angiotensin II blockers, hyperlipidemia with statins, and smoking^5^. The interference against the glycation end-products and its receptor could be a therapeutic strategy for progression prevention.

After diagnosis, SHaNGS has a poor prognosis for renal function. It has been documented that the median time from biopsy to end stage renal disease (ESRD) is 26 months and 23.5% of patients will require renal replacement therapy at a mean of 8.7 months¹. The risk factors for ESRD are continuation of smoking (p 0.0165), no angiotensin II blocker use (p 0.0007), advance tubular atrophy and interstitial fibrosis (p. 0517), and advance arteriosclerosis (p 0.0096)^1^. In contrast, patients with an initial serum creatinine <3.0 mg/dL showed slower progression rate to ESRD with angiotensin II blockers use (p 0.0126) and patients who stop smoking or never smoked did not reach ESRD^1^ immunohistochemical profiles, and outcomes in 23 patients with ING diagnosed from among 5,073 native renal biopsy samples (0.45%.

SHaNGS or ING is a rare disease which has a wide diagnostic approach, thus the importance of analyzing every piece of detail together to reach a definitive diagnosis is highlighted.
